# Cystic fibrosis therapy: from symptoms to the cause of the disease

**DOI:** 10.18699/vjgb-25-31

**Published:** 2025-04

**Authors:** T.N. Kireeva, D.I. Zhigalina, N.A. Skryabin

**Affiliations:** Tomsk National Research Medical Center of the Russian Academy of Sciences, Tomsk, Russia; Tomsk National Research Medical Center of the Russian Academy of Sciences, Tomsk, Russia; Tomsk National Research Medical Center of the Russian Academy of Sciences, Tomsk, Russia

**Keywords:** cystic fibrosis (CF), CFTR, CFTR mutations, CFTR modulators, gene therapy, genome editing, CRISPR/Cas9, муковисцидоз (МВ), ген CFTR, мутации CFTR, модуляторы CFTR, генная терапия, геномное редактирование, CRISPR/Cas9

## Abstract

Cystic fibrosis (CF) is a disease with a broad clinical and genetic spectrum of manifestations, significantly impacting the quality and duration of life of patients. At present, a diagnosis of CF enables the disease to be identified at the earliest stages of its development. The accelerated advancement of scientific knowledge and contemporary research techniques has transformed the methodology employed in the treatment of CF, encompassing a spectrum of approaches from symptomatic management to pathogenetic therapies. Pathogenetic therapy represents an approach to treatment that aims to identify methods of restoring the function of the CFTR gene. The objective of this review was to analyse and summarize the available scientific data on the pathogenetic therapy of CF. This paper considers various approaches to the pathogenetic therapy of CF that are based on the use of targeted drugs known as CFTR modulators. The article presents studies employing gene therapy techniques for CF, which are based on the targeted delivery of a normal copy of the CFTR gene cDNA to the respiratory tract via viral or non-viral vectors. Some studies have demonstrated the efficacy of RNA therapeutic interventions in restoring splicing, promoting the production of mature RNA, and increasing the functional expression of the CFTR protein. The review also analyzes literature data that consider methods of etiotropic therapy for CF, which consists of targeted correction of the CFTR gene using artificial restriction enzymes, the CRISPR/Cas9 system and a complex of peptide-nucleic acids. In a prospective plan, the use of cell therapy methods in the treatment of lung damage in CF is considered.

## Abstract (ru)

Муковисцидоз (МВ) – заболевание с широким клиническим и генетическим спектром проявлений, оказывающее значительное влияние на качество и продолжительность жизни пациента. В настоящее время диагностика МВ позволяет выявлять заболевание на самых ранних стадиях. Стремительное развитие науки и современные методы исследования изменили подходы в лечении МВ, начиная от симптоматического лечения до методов патогенетической терапии. Подходы патогенетической терапии направлены на поиск способов восстановления функции гена CFTR. Целью обзора стали анализ и обобщение имеющихся научных сведений о патогенетической терапии МВ. Рассмотрены подходы патогенетической терапии МВ на основе приема пациентами таргетных препаратов – CFTR-модуляторов. Приведены исследования с использованием методов генной терапии МВ, в основе которых лежит целенаправленная доставка нормальной копии кДНК гена CFTR в дыхательные пути с помощью вирусных или невирусных агентов. В некоторых исследованиях показано применение методов РНК-терапии для восстановления сплайсинга, продукции зрелой РНК и функционального белка CFTR. Также в обзоре проведен анализ литературных данных, в которых рассмотрены методы этиотропной терапии МВ, заключающейся в направленной коррекции гена CFTR с использованием искусственных ферментов рестрикции, системы CRISPR/Cas9 и комплекса пептидно-нуклеиновых кислот. В перспективном плане обсуждаются методы клеточной терапии в лечении поражения легких при муковисцидозе.

## Introduction

Cystic fibrosis (CF) (OMIM 219700) is a monogenic orphan disease with autosomal recessive type of inheritance, systemic organ damage with a severe course of the disease and prognosis (https://www.omim.org/). The incidence of CF is on average one case per 2,500–3,000 newborns (Kashirskaya, Kapranov, 2014). CF is most frequently registered among Caucasians, for example, in the USA and Europe, there are about 70,000 patients with CF; in Russia, there are about 4,000 patients with this disease (Simonova et al., 2020; Lomunova, Gershovich, 2023).

Cystic fibrosis is caused by pathogenic variants in the cystic fibrosis transmembrane conductance regulator (CFTR) gene. The CFTR gene was identified and cloned in 1989 (Gembitskaya et al., 2012; Elborn, 2016; Spielberg, Clancy, 2016). The CFTR gene contains 27 exons and is located in region 31.1 of the long arm of chromosome 7 (7q31.1). The protein encoded by this gene, the CFTR transmembrane conductance regulator, is a member of the ABC transporter (ATP-binding cassette) superfamily of proteins. The structural organization of the CFTR protein includes two transmembrane domains (TMD1 and TMD2), two nucleotide- binding domains (NBD1 and NBD2), and a central intracellular regulatory domain (R-domain). Localized in the apical membrane of epithelial cells, the CFTR protein creates a chloride channel regulated by cyclic adenosine monophosphate (cAMP). The CFTR protein regulates not only chloride ions (Cl−), but also bicarbonate ( H C O −3 ) secretion, which regulates the pH of the fluid on the surface of airway cells. CFTR also plays an important role in the hydration of secretions and mucins through inhibition of the epithelial sodium channel (ENaC) (Ginter, 2000; Moran, 2014; Kondratieva et al., 2018; Bell et al., 2020; Hanssens et al., 2021).

Mutations in the CFTR gene lead to disruption of ion channels, causing a decrease in conductivity for Cl− ions and an increase in conductivity for Na+ ions. These disorders cause changes in hydration processes in the apical membrane of epithelial cells and changes in the viscoelastic properties of substances produced by exocrine glands. These changes have a greater impact on the functioning of the respiratory system, pancreas, liver, bile ducts, gastrointestinal tract, sweat glands and organs of the male reproductive system (Dechecchi et al., 2018; Smirnikhina, Lavrov, 2018; Lomunova, Gershovich, 2023).

## CFTR gene variants

The identification and characterization of CFTR gene variants is carried out by the international Cystic Fibrosis Genetic Analysis Consortium (CFGAC), which unites laboratories, the activities of which are aimed at genetic diagnostics and research of CF around the world. For general access, the obtained data are placed in the database of CFTR gene variants “CFTR1” (http://www.genet.sickkids. on.ca/cftr/) and the subsequently created database “CFTR2” (http://www.cftr2.org/). The CFTR2 database includes current information on recently discovered CFTR gene variants (Rommens et al., 2006; Dechecchi et al., 2018; Kondratieva et al., 2018). Currently, the CFGAC database contains more than 2,000 variants in the CFTR gene, which are divided into seven classes, depending on the mechanism of their effect on the function of the CFTR protein (Fanen et al., 2014; Elborn, 2016; Kondratieva et al., 2018; Bell et al., 2020; Lee et al., 2021; Krasnova et al., 2023).

In class I of genetic variants (R553X, W1282X, 2143delT, G542X, 1677delTA), there is no functional CFTR protein, resulting in impaired transcription and translation. Approximately 22 % of patients with CF have at least one mutant allele of this class (Lee et al., 2021). As a result of genetic variants of class II (F508del, I507del, N1303K, S549N), the maturation of the CFTR protein is blocked due to an incorrect configuration of its molecule. Misfolded protein molecules do not reach the surface of epithelial cells because they undergo endoplasmic reticulum-associated protein degradation (ERAD). Approximately 88 % of CF patients have at least one mutant allele and the main variant is F508del, caused by a deletion of phenylalanine at position 508. With the genetic variant F508del, there is a disruption of post-translational modification of the CFTR protein, which leads to the protein molecule becoming functionally defective and unstable, or being completely destroyed (Van Goor et al., 2006; Smirnikhina, Lavrov, 2018).

Genetic variants of class III (G1224E, S1255P, G551D) which are localized in the regulatory domain of the CFTR protein and its nucleotide binding domains, lead to a disruption in the regulation of the chloride channel. The defect in the chloride channel in this case is due to the fact that the CFTR protein is synthesized and transported to the cell membrane, but does not respond to cAMP stimulation. Missense mutations, related to genetic variants of class IV in the CFTR gene, (R117H, R347P, R334W) lead to a decrease in ion flow as a result of changes in the conductivity of the chloride channel. These variants are located in transmembrane domains and affect the reduction of ion channel opening time. About 6 % of patients with CF have this type of genetic variant. CFTR gene variants of class V reduce the levels of functional protein and its transport to the apical membrane surface, which is characteristic of 5 % of patients with CF. Class VI includes CFTR gene variants that alter protein stability, resulting in a decrease in the time the protein remains on the membrane surface. It has been noted that 5 % of patients with CF have at least one allele of this variant (Kondratieva et al., 2018; Dechecchi et al., 2018). Class VII is also distinguished: its genetic variants affect the expression of CFTR protein mRNA. The absence of mRNA is caused by a genetic variant characterized by a large deletion – CFTRdele2,3 (21 kb) (Lee et al., 2021).

It is important to start therapy in a timely manner to prevent the development of severe complications in CF and generally improve the prognosis of the disease. The results of fundamental research have allowed us to expand our understanding of the main pathogenetic and pathophysiological mechanisms of CF, which contributed to the rapid development and emergence of new approaches in the treatment of this disease. Currently, the basis of treatment for patients with CF is complex therapy, combining methods of both symptomatic and pathogenetic treatment. Methods based on the use of tools for CFTR gene correction are also being considered in the future (Gembitskaya et al., 2012; Bell et al., 2020).

## Symptomatic treatment of CF

Symptomatic treatment is aimed at combating infection, improving mucus clearance from the bronchi and preventing nutritional deficiencies, including macro- and micronutrient deficiencies. Patients with CF are prescribed antibiotics, mucolytic and bronchodilator drugs in combination with enzymes, vitamins and a course of kinesiotherapy (Kashirskaya, Kapranov, 2014; Simonova et al., 2020). To treat respiratory lesions in patients with CF, anti-inflammatory and massive antibacterial therapy is used, while it is noted that the inhalation route of drug administration (mucolytics, bronchodilators, antibiotics and glucocorticoids) is highly effective (Gembitskaya et al., 2012; Olveira et al., 2017; Kondratieva et al., 2018; Simonova et al., 2020).

Methods of optimized antibiotic therapy have a significant impact on the course of CF, where the choice of antibiotic depends on the microbiological status of the patient. Antibiotic resistance is overcome by aerosol delivery of antibiotics into the bronchial lumen, which also reduces side effects during long-term treatment and the use of high doses, since the concentration of drugs in the blood serum is low in this case (Gorinova et al., 2015; Kondratieva et al., 2018; Simonova et al., 2020).

In the treatment of CF, mucolytic drugs are prescribed to normalize the viscous-elastic properties of sputum and improve its transport. In this group of drugs, a great advantage is possessed by the genetically engineered drug – the mucolytic dornase alpha, which has a complex effect on the infection, inflammation and obstruction observed in CF. The use of this drug is of great importance in the complex treatment of the bronchopulmonary process in patients with CF, especially immediately after diagnosis (Sherman et al., 2011). Together with mucolytic drugs, patients with CF are prescribed special active breathing exercises (kinesiotherapy) to remove phlegm from the respiratory tract (Simonova et al., 2020).

No less important in the therapy of CF is the correction of exocrine pancreatic insufficiency and treatment of hepatobiliary disorders, as well as maintaining the nutritional status of patients with the help of diet therapy. In patients with pancreatic insufficiency, in addition to diet therapy, enzyme replacement therapy and fat-soluble vitamins are also prescribed (Kashirskaya, Kapranov, 2011, 2014; Kondratieva et al., 2018).

All the developed methods and applied drugs for symptomatic treatment affect not only the life expectancy of patients with CF, but also their quality of life, significantly improving it. However, symptomatic treatment is aimed only at controlling symptoms and limiting complications in CF, while not affecting the functioning of the defective CFTR protein in any way (Smirnikhina, Lavrov, 2018; Simonova et al., 2020).

## Pathogenetic therapy of CF

The development and testing of new methods and drugs aimed at finding ways to restore the function of the CFTR gene is becoming relevant. In this direction, pathogenetic therapy methods are considered promising (Gembitskaya et al., 2012; Rafeeq, Murad, 2017; Bell et al., 2020). Given the diversity of genetic variants in the CFTR gene and their various clinical manifestations, studies have been conducted to find drugs that suppress premature termination of protein translation for patients with nonsense mutations of class I, drugs for carriers of the common F508del variant and other genetic variants of class II, as well as drugs that work with all classes of genetic variants (see the Table) (Kondratieva et al., 2018; Dechecchi et al., 2018).

**Table 1.**
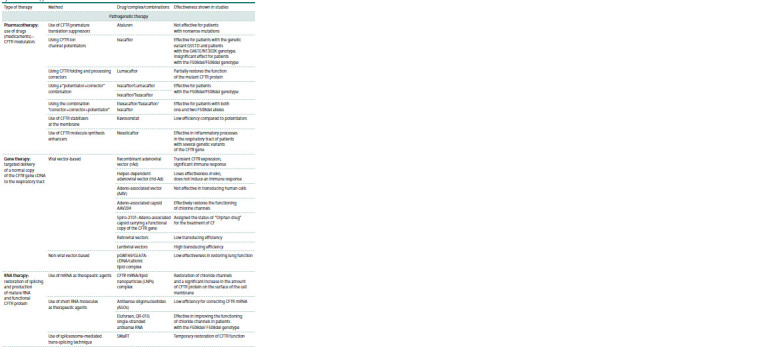
Cystic fibrosis therapy

**Table 1end.**
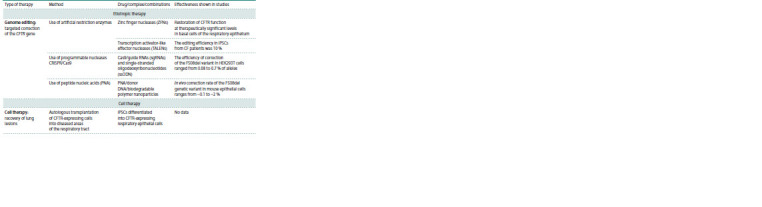
Table (end)

The most promising therapeutic agents for the treatment of CF turned out to be a group of modulators, which are small molecule drugs that were identified as a result of high-throughput screening to correct impaired CFTR protein transport to the plasma membrane or to increase the conductance of the chloride channel (Dechecchi et al., 2018; Sui et al., 2022; Krasnova et al., 2023). In CF therapy, the choice of modulator drug depends on the class of CFTR gene variant and the direction of their compensatory actions. In this regard, modulators are divided into potentiators, correctors, amplifiers and stabilizers (Lee et al., 2021).

## Potentiators

The action of potentiators is aimed at enhancing the opening of the ion channel formed by the mutant CFTR protein on the cell surface. The effect on the ion channel is achieved through activation of the adenylate cyclase pathway (CFTR gene variants of classes III–IV). Ivacaftor (Vertex Pharmaceuticals, Germany) is one of the drugs in this group. Phase I trials of ivacaftor were conducted in healthy volunteers and showed the safety of the drug (Van Goor et al., 2009). Then, in 2011, based on the results of tests on 112 CF patients (USA), data were presented that in individuals with the G551D, G178R, G551S, G1244E, G1349D mutations, a reliable increase in the transport of chloride ions was established (Flume et al., 2012). In 2012, Food and Drug Administration (FDA) approved ivacaftor for use in patients with at least one out of 38 point mutations, including five splicing mutations (Van Goor et al., 2006, 2009; Smirnikhina, Lavrov, 2018). Ivacaftor is recommended worldwide, including in Russia, for patients with the G551D mutation (Sui et al., 2022). In 2017, a case of successful treatment of a patient with CF with the G461E/N1303K genotype was described; after six months of using ivacaftor, the patient’s clinical course of the disease changed significantly (Amelina et al., 2017). Since 2018, data have been published evaluating the efficacy of ivacaftor in a group of pediatric patients (2–3 years old), where more preserved lung function and a lower level of complications observed in CF were noted (Bessonova et al., 2018).

## Correctors

Correctors are pharmacological substances that bind to the mutant CFTR protein, promoting its “maturation” by adapting protein homeostasis and reducing the degradation of the mutant protein in the intracellular quality control system (CFTR gene variants of class II) (Smirnikhina, Lavrov, 2018). Among the group of corrective drugs, the use of 4-phenylbutyrate/ genistin, curcumin, tezacaftor, and lumacaftor is known. The largest number of studies is devoted to the evaluation and analysis of the effectiveness of lumacaftor in stabilizing the mutant CFTR protein and its movement from the endoplasmic reticulum (ER) to the surface of the cell membrane. Moreover, it was shown that lumacaftor is able to partially restore the function of the mutant CFTR protein by stabilizing its N-terminal domain (Ren et al., 2013; Lee et al., 2021).

It was later shown that the use of lumacaftor or ivacaftor alone only slightly reduced sweat chloride levels for patients homozygous for the F508del mutation. This suggests that monotherapy with either modulator is ineffective in improving lung function (Flume et al., 2012; Hanssens et al., 2021). Subsequently, the effectiveness of various combinations of modulators was assessed to restore CFTR protein function in patients with the F508del/F508del genotype. A number of larger studies have shown that long-term combination therapy with lumacaftor and ivacaftor was effective in patients over 12 years of age who were homozygous for the F508del mutation (Boyle et al., 2014; Wainwright et al., 2015). For Russian patients, a combination of lumacaftor and ivacaftor is used in the treatment of CF together with basic therapy, which results in such improvements as a decrease in the level of chlorides in sweat fluid, an increase in the forced expiratory volume in 1 second (FEV1), an improvement in the general condition and weight gain (Amelina et al., 2019).

As a result of the fact that the combination of a potentiator and a corrector has a positive effect on the clinical effect in patients with the F508del mutation, in 2015, the FDA approved the combination drug lumacaftor/ivacaftor for use in the treatment of CF. This drug is approved for use in children over 6 years of age and adults with the F508del/ F508del genotype (Dechecchi et al., 2018; Smirnikhina, Lavrov, 2018; Simonova et al., 2020). Despite the efficacy demonstrated in clinical trials, the use of this drug entails a number of side effects, and, in addition, a positive effect is observed only in the case of one genetic variant, F508del, which is in a homozygous state (Lee et al., 2021).

The combination of ivacaftor with another drug, tezacaftor, has shown a positive therapeutic effect in improving lung function in the treatment of patients who are homozygous for F508del. The combination drug tezacaftor+ivacaftor/ ivacaftor is used to treat CF in children 12 years of age and older and adults with the homozygous F508del mutation (Taylor-Cousar et al., 2017).

According to Vertex, a combination of three new-generation modulators, elexacaftor/tezacaftor/ivacaftor (ETI), has shown the greatest effectiveness in treating patients with the F508del/F508del genotype (Smirnikhina, Lavrov, 2018). This combination drug increases the activity of the CFTR protein and reduces mortality and morbidity in patients with CF, and is applicable both to CF patients homozygous for F508del (in 90 % of cases) and to the group of patients heterozygous for F508del and the variant with residual function (Keating et al., 2018). In clinical studies, the use of the ETI combination has been shown to improve mutant CFTR protein function to levels of 40–50 % of normal CFTR protein activity in airway and intestinal epithelial cells. This combination has also been shown to be highly effective in improving lung function, reducing sweat chloride concentration, and reducing pulmonary exacerbation frequency (Piehler et al., 2023).

## Stabilizers and amplifiers

In the treatment of patients with CF, it is necessary to use compounds that stabilize and enhance the CFTR protein. By fixing the CFTR protein to the plasma membrane, stabilizers prevent its detachment and degradation in lysosomes. Nivalis Therapeutics has developed a compound that stabilizes the protein, cavosonstat, which was clinically tested in 138 patients homozygous for F508del. Patients received cavosonstat in combination with ivacaftor. However, in phase II, this study was completed due to the lack of advantages of the stabilizer compared to potentiators (Krasnovidova et al., 2023).

Enhancers are used to increase the amount of CFTR protein molecules synthesized in cells, available for subsequent modulation by protein-active small molecules. This group includes the drug nesolicaftor, which was developed by Proteostasis Therapeutics. Nesolicaftor enhances CFTR synthesis and, in combination with other existing CF treatments, has shown a positive effect on protein activity in vitro, nearly doubling its activity in bronchial epithelial cells of patients with multiple genetic variants of the CFTR gene. When using nesolicaftor in combination with ETIs in primary human bronchial epithelial F508del cells, it was shown to reverse cytokine transforming growth factor beta 1 (TGF- β1)-mediated inhibition of corrected CFTR function, likely through mRNA stabilization. Nesolicaftor also indirectly increases the level of secreted cytokines through its effect on apical ion channel function. The use of enhancers has been shown to be effective in treating inflammatory processes in the airways of patients with CF (Bengtson et al., 2022).

Thus, the considered pharmacological agents for pathogenetic therapy of CF significantly increased the life expectancy of patients with this diagnosis. However, CFTR modulators do not eliminate the cause of the disease, but only correct the functioning of the defective protein. CFTR modulator therapy requires lifelong drug administration, and their long-term potential side effects remain unclear (Sui et al., 2022). It is also noted that approximately 10 % of patients are resistant to modulators due to the absence or low levels of the CFTR protein. Also, according to clinical studies, about 10–20 % of patients with CF have individual intolerance to modulator drugs (Smirnikhina, Lavrov, 2018; Lee et al., 2021; Lomunova, Gershovich, 2023). In this regard, new methods of treating CF are being developed, aimed at eliminating the pathological changes underlying the development of this disease. First of all, these are gene therapy methods (Maule et al., 2020).

## Gene therapy for CF

The monogenic and recessive type of inheritance in CF has led to the emergence of treatment methods for this disease using gene therapy methods (see the Table) (Sui et al., 2022). Gene therapy for CF involves the targeted delivery of a normal copy of complementary DNA (cDNA) of the CFTR gene to the most affected areas of the respiratory tract of patients using viral particles carrying the target transgene and non-viral agents, such as liposomes, nanoparticles, etc. (Ginter, 2000; Smirnikhina, Lavrov, 2018; Lomunova, Gershovich, 2023).

In 1993, a study was initiated to deliver a normal copy of CFTR cDNA to the nasal epithelium of CF patients using a recombinant adenoviral vector (rAd). This study demonstrated the potential of using recombinant adenoviral vectors to temporally correct Cl– ion transport in CF. However, it was subsequently shown that rAd-mediated CFTR expression in postmitotic airway epithelial cells is transient and promotes robust cellular and humoral immune responses (Van Goor et al., 2009). Subsequently, a helper-dependent adenoviral vector (Hd-Ad) was developed to eliminate the problem of immune response. Hd-Ad delivers DNA (up to 37 kb) to airway cells, excluding host T cell responses to the expression of foreign viral protein, i. e. without causing inflammation (Lee et al., 2021). A study in CF mouse and pig airway basal cells showed restoration of CFTR function to levels seen in normal wild-type cells after correction of CFTR with Hd-Ad. In lung cells from CFTR gene knockout mice, the effectiveness of Hd-Ad vectors for CFTR gene correction was also demonstrated. However, due to airway cell turnover, the use of Hd-Ad vectors in vivo for CFTR gene correction loses its therapeutic efficacy (Koehler et al., 2003; Cao et al., 2020).

From 1998 to 2007, clinics led by Targeted Genetics Corporation evaluated the potential of using adeno-associated vectors (AAV) in the treatment of CF lung disease, of which rAAV2 was the only available vector of this serotype. Preclinical studies have demonstrated the ability of rAAV2 to productively transduce lung cells from rhesus macaques and rabbits. However, more recent studies of rAAV2 transduction biology in a polarized human airway epithelium (HAE) cell culture model at the air-liquid interface (ALI) have found that rAAV2 poorly transduces human airway epithelial cells. Another limitation of the use of rAAV vectors in CFTR gene transfer is their relatively small packaging capacity (~4.9 kb) (Sui et al., 2022).

In recent years, several pharmaceutical companies have been developing AAV-based gene therapy agents. For example, Abeona Therapeutics has developed a next-generation capsid, AAV204, which carries a functional copy of the human mini-CFTR gene. The use of this agent in therapy allows for the effective restoration of the functioning of chloride channels in cells, both in vitro and in vivo. In 2020, Spirovant Sciences introduced another adeno-associated capsid with improved tropism for airway epithelial cells for delivering a functional copy of the CFTR gene. FDA granted Spirovant Sciences Orphan Drug Designations for Spiro-2101 for Treatment of CF (Lee et al., 2021; Lomunova, Gershovich, 2023).

Retroviral and lentiviral vectors have also been shown to be useful in CF gene therapy. In studies on rabbits, the use of retroviruses carrying the CFTR gene demonstrated persistent expression of this gene in their respiratory tract for up to three weeks, but low transduction efficiency was observed (Lee et al., 2021). The advantage of lentiviral vectors derived from immunodeficiency viruses is their ability to transduce both dividing and non-dividing cells, and transgene expression from the integrated viral genome is likely to be maintained throughout the life cycle of recipient cells. In this case, lentiviral vectors used for transduction into respiratory epithelial cells must be pseudotyped with appropriate protein coats. Studies have shown higher transduction efficiency into airway cells using a lentiviral strategy compared to a non-viral one (Alton et al., 2015; Sui et al., 2022). However, non-viral methods of delivering the normal CFTR gene are safer and better tolerated due to the absence of insertional mutagenesis and secondary effects of altered transgene expression levels (Lee et al., 2021).

Another advantage of using non-viral vectors is the ability to use larger fragments of donor DNA for gene repair. For efficient non-viral delivery of CFTR, a cDNA/cationic lipid complex is used. According to a study published by the UK CF Gene Therapy Consortium, CFTR function increased by up to 3.7 % in lung cells from CF patients after treatment with the nebulized cationic lipid pGM169/GL67A, which delivers donor DNA from the normal CFTR gene. However, this improvement was still not sufficient to restore lung function in CF (Alton et al., 2015; Spielberg, Clancy, 2016).

Thus, for almost three decades now, the search for suitable gene therapy methods for the treatment of CF has been ongoing. There have been approximately 36 clinical trials of gene therapy involving a significant number of patients with CF; however, due to the low clinical effect, these studies have not been further developed. Nevertheless, these studies have shown the promise of the concept of gene therapy for CF and have created a great foundation in this field (Sui et al., 2022).

## CFTR gene editing

New approaches to targeted gene correction have come into use thanks to the emergence and improvement of experimental cellular and animal models. One of these effective methods is genome editing methods (see the Table).

To correct genes, tools are used based on targeted DNA cleavage using artificial restriction enzymes: zinc-finger nucleases (ZFNs), transcription activator-like effector nucleases (TALENs) and with the help of a programmable nuclease (most often Cas9), the specificity of which is achieved using guide RNA (sgRNA). The mechanisms functioning in the cell – non-homologous end joining (NHEJ) and homologous-directed repair (HDR), a common form of which is homologous recombination (HR), – ensure DNA repair (Smirnikhina et al., 2020; Lee et al., 2021).

Using ZFNs, the feasibility of editing the CFTR locus in airway basal cells derived from CF patients was assessed using two approaches. The first approach, based on sequence replacement to correct F508del, demonstrated restoration of mature CFTR protein and its function in ALI border cultures derived from massively edited basal cells. The second approach aimed to integrate partial cDNA into an intron of the endogenous CFTR gene to correct all genetic variants of the CFTR gene. As a result, highly efficient site-specific targeted integration into basal cells harboring different genetic variants of the CFTR gene was observed and restoration of CFTR function at therapeutically relevant levels was demonstrated (Suzuki et al., 2020). The use of TALEN in experiments shows better affinity than ZFNs. In one study, Hd-Ad vectors were used to deliver TALENs with donor DNA into cells, resulting in approximately 5 % targeted gene integration. TALEN-mediated editing of F508del has also been demonstrated in induced pluripotent cells (iPSCs) derived from CF patients, with editing efficiency in this case being no greater than 10 %. It was noted that manipulations with the iPSC genome did not affect their properties and ability to differentiate (Holkers et al., 2013; Xia et al., 2019).

Genome editing using CRISPR/Cas9 (Clustered Regulatory Interspaced Short Palindromic Repeats/CRISPR associated protein 9) allows editing a pathogenic variant in a gene with high efficiency and allows fixing the “corrected” allele in the genome. CRISPR/Cas9 is a very promising technology for creating valuable experimental tools for testing treatments for a wide range of pathogenetic variants that cause CF (Smirnikhina, Lavrov, 2018). The first use of the CRISPR/Cas9 editing system to correct the CFTR gene locus was applied in cultured intestinal stem cells from patients homozygous for F508del. The genetically modified stem cells formed organoids that responded functionally to forskolin through changes in volume. In another study, iPSCs were generated from fibroblast cells from CF patients (F508del), which were also subsequently modified to contain the CFTR gene using the CRISPR/Cas9. Corrected iPSCs were able to differentiate into mature airway epithelial cells and demonstrated restoration of chloride transport (Wang, 2023).

To develop methods for editing genetic variants in the CFTR gene, a variety of cell lines have been generated using Cas9 nucleases, representing alternative models. These models are cell cultures into which plasmids carrying synthetic vectors with a fragment of the CFTR gene containing a target mutation, including a rare one, have been introduced. Based on this approach, the following cell lines were created: human lung cancer (Calu-3 CF), human leukemia (HL-60 F508del-CF), human carcinoma (T84 F508del-CF), human bronchial epithelial cells (16HBE14o-CF with F508del), as well as isogenic cell models with the G542X, W1282X mutations (Wang, 2023). A model of CF was created in HEK293T cell culture by introducing the synthetic plasmid pGEM-CFTR, carrying the CFTR locus with the F508del mutation. The efficiency of correction of this genetic variant was then assessed using six different combinations of Cas9/guide RNA (sgRNA) and single-stranded oligodeoxyribonucleotides (ssODN). The efficiency of correction of the F508del mutation ranged from 0.08 to 0.7 % of alleles, depending on the combination of CRISPR/Cas9 components used (Smirnikhina et al., 2020).

In addition to the considered editing systems, the possibility of correcting the CFTR gene was demonstrated using peptide nucleic acids (PNA) not based on CRISPR. In studies on F508del airway epithelial cells, triplex-forming peptide nucleic acids and donor DNA packaged in biodegradable polymer nanoparticles were used. The results show that intranasal delivery of nanoparticles to CF mice induces changes in the nasal epithelial potential difference assay as a consequence of corrected CFTR function. Another study demonstrated in vivo correction of the F508del mutation in multiple epithelial cells, including nasal epithelium, trachea, lung, ileum, colon and rectum in CF mice with systemic delivery of PNA. The correction level ranged from ~0.1 to ~2 % (Wang, 2023).

The approaches considered in targeted correction of the CFTR gene are aimed at the causes underlying the disease, i. e. they have the potential to provide a permanent cure for patients with CF. Despite this significant advantage, these approaches are currently not used in clinical practice due to bioethical restrictions.

## RNA therapy for CF

In the therapy of CF, the use of methods based on the use of RNA is considered: messenger RNA (mRNA), transfer RNA (tRNA) and smaller RNA molecules – oligonucleotides, as therapeutic agents (see the Table). Clinical trials are currently underway investigating the potential of mRNA in CF therapy. The RESTORE-CF study (NCT03375047) tested specialized lipid nanoparticles (LNPs) as mRNA carriers. The results of these tests are measured by changes in lung function, i. e. changes in FEV1. After introducing chemically modified CFTR mRNA into cells using relevant liposomal nanoparticles, restoration of the functioning of chloride channels and a significant increase in the amount of CFTR protein on the surface of the cell membrane of the respiratory epithelium of patients with CF were noted (Lomunova, Gershovich, 2023).

In order to restore splicing and production of mature RNA and functional CFTR protein, the use of antisense oligonucleotides (ASOs) is being considered (Egan, 2021). More than 40 clinical trials have been conducted to study the therapeutic potential of ASOs in the treatment of CF. In cell models with the F508del genetic variant, ASO was used to insert missing bases at position 508 of CFTR at the RNA transcript level, but this method of mRNA correction was not stable (Maule et al., 2020). ProQR Therapeutics conducted studies on intranasal administration of single-stranded antisense RNA (eluforsen, QR-010) to mice. This drug was designed to restore CFTR function in respiratory epithelium through specific binding to the F508del region of mRNA. Studies have shown that QR-010 successfully diffuses into cells and causes positive changes in chloride transport. Thus, after three intranasal administrations of QR- 010 over four weeks, patients with F508del/F508del showed a clinically significant improvement in the functioning of the chloride channel due to the restoration of CFTR function (Lomunova, Gershovich, 2023).

Spliceosome-mediated RNA trans-splicing (SMaRT) was also used to restore nascent mRNA by replacing part of the altered transcript with the correct exogenous mRNA. This technique was used in cell models with F508del to restore correct transcripts. However, this method only temporarily restored CFTR function (Maule et al., 2020).

The RNA therapies discussed above are considered possible treatments for patients with CF; however, these treatments require lifelong administration of therapeutic agents, as does CFTR modulator therapy.

## Cell therapy for CF

In the future, the use of cell therapy methods in the treatment of lung damage in CF is being considered (see the Table). However, the method of delivering donor cells to human lungs poses significant difficulties.

Experiments on mice have shown the possibility of delivering cells to their lungs, e. g. embryonic stem cells (ESCs) were introduced into the lungs of mice by intravenous administration, and bone marrow (BM) cells were introduced by intratracheal administration. However, in these cases, the efficacy was low (Lee et al., 2021). Several studies have been conducted on the introduction of multipotent mesenchymal stromal cells (MMSCs) into the affected lungs of mice, where it was shown that the introduction of intact MMSCs into the body activates anti-inflammatory immunity in animals with various forms of lung inflammation (Smirnikhina, Lavrov, 2018). A study conducted at Stanford University involved editing the mutant CFTR gene in primary airway basal cells using the CRISPR/Cas9 system delivered to these cells using AAV vectors. The corrected basal cells were then placed into rat sinus cavities, where the ability of these cells to proliferate into CFTR-normal cells was further assessed (Egan, 2021).

To date, protocols for differentiation of iPSCs into CFTRexpressing respiratory epithelial cells have already been developed, which allows iPSCs to be considered a promising material for autogenous transplantation in lung lesions. However, at present, clinical trials using iPSCs as part of cell therapy for patients with CF are not being conducted (Lomunova, Gershovich, 2023).

## Conclusion

The ultimate goal of research into the discovery and development of treatments for CF is to provide all patients with therapy early enough in life to delay or even prevent many of the disease’s manifestations, and to personalize the overall therapy itself based on patients’ needs.

The advent of a number of targeted drugs in 2012 gave rise to a personalized approach to the treatment of patients with CF. Some drugs have already passed clinical trials and are used in therapy; these drugs include first-generation CFTR modulators: ivacaftor, lucamaftor/ivacaftor, teza-caftor+ ivacaftor/ivacaftor, elexacaftor/tezacaftor/ivacaftor+ ivacaftor. The use of modulators in CF therapy has made it possible to restore the functions of the mutant CFTR protein and improve the functioning of chloride channels on the surface of cells. However, this modulator therapy is not curative and does not cover all mutations in the CFTR gene. For the 10 % of CF patients with missense mutations, where cells produce little to no CFTR protein, therapy with CFTR modulators is not an option, making research into CF gene therapy, including genome editing, of great importance.

The advantage of gene therapy is that it is suitable for all CF patients, regardless of their genotype. There have been large research programs in the area of gene therapy for CF, developing potential agents for this type of therapy, and numerous clinical trials have been conducted to deliver the normal CFTR gene into respiratory epithelial cells. However, the long road to using gene therapy as a treatment for CF has not resulted in significant consistent clinical efficacy, even though there may have been some level of correction. Approaches using methods of genomic editing of the CFTR gene in CF are considered, using such tools as CRISPR/ Cas9, ZFNs, TALEN and peptide nucleic acids. Research on genome editing in CF is in the preclinical phase.

Thus, patients with CF have been given the opportunity to significantly increase their life expectancy, along with improving its quality, thanks to the huge amount of research into the pathogenesis of CF and developments using innovative gene-directed personalized treatment methods.

## Conflict of interest

The authors declare no conflict of interest.
